# 

**Published:** 1848-10

**Authors:** 


☞ Subscribers, out of the city, indebted for this Journal, are urgently requested to remit their dues before the first of November. City subscribers are respectfully notified that they will soon be waited upon by a collector.

The Review of M'Lellan's Surgery will be concluded in our next number.

---

*Buffalo Medical College.*—The preliminary dissecting term in this Institution will commence on the last Wednesday of the present month.

